# Integrated transcriptomic profiling combined with *in vitro* validation reveals the involvement of TMEM140 in the link between periodontitis and brain aging

**DOI:** 10.3389/fnagi.2026.1761218

**Published:** 2026-04-22

**Authors:** HaoRan Zhao, HongTao Wang, WangXing Li, Rui Su, Jing Li, Yan Liu, Lei Wang

**Affiliations:** 1 Kunming Medical University Haiyuan College, Kunming, Yunnan, China; 2 Department of Cardiovascular Surgery, Kunming Yan’an Hospital, Kunming, Yunnan, China; 3 Neuroscience Research Institute, Kunming Medical University, Kunming, Yunnan, China

**Keywords:** AD, aging, Alzheimer’s disease, PD, periodontitis, TMEM140

## Abstract

**Objective:**

Periodontitis (PD) is a prevalent chronic inflammatory disorder in adults, and moderate-to-severe PD (Stage II-III/IV) may accelerate brain aging and neurodegenerative changes via the peripheral-central immune-neural axis, although the molecular connections and mechanisms of interaction have yet to be fully elucidated. This study sought to identify senescence-associated molecules potentially shared by PD and Alzheimer’s disease (AD) using integrated transcriptomic analysis, machine learning, and *in vitro* RNA interference assays, and to further assess the role of TMEM140 in linking PD to brain aging.

**Methods:**

Transcriptomic datasets related to PD and AD were retrieved from the GEO database, and differential gene expression analysis was performed following batch effect correction; shared aging-associated genes were subsequently identified by combining weighted gene co-expression network analysis (WGCNA) with aging gene databases (HAGR and aging Atlas). Four machine learning algorithms, namely random forest (RF), support vector machine (SVM), generalized linear model (GLM), and extreme gradient boosting (XGB), were further applied to identify key genes, and their diagnostic value was assessed using receiver operating characteristic (ROC) analysis and nomogram models. DSigDB was used to predict candidate small-molecule compounds. In the *in vitro* experiments, a *Porphyromonas gingivalis* lipopolysaccharide (PG-LPS)-induced inflammatory model in human gingival fibroblasts (HGFs) and an Aβ1-42 and D-galactose-induced senescence model in SH-SY5Y neuron-like cells were established; TMEM140 in SH-SY5Y cells was then silenced using small interfering RNA (siRNA), and the neuron-like cells were treated with the same batch of standardized conditioned medium (CM; prepared from the supernatant of PG-LPS-treated HGFs) to observe changes in cellular responses to inflammatory stimulation after TMEM140 downregulation.

**Results:**

Seven aging-related genes common to PD and AD were identified, and comprehensive analysis using multiple algorithms selected TMEM140, TIMP1, and ALDH2 as key genes. Notably, TMEM140 was upregulated in PD and downregulated in AD, showed significant correlations with plasma cell and γδ T-cell infiltration, and single-cell analysis further revealed its cell type-specific expression in distinct brain cell subsets. *In vitro* experiments demonstrated that PG-LPS treatment markedly increased TMEM140 expression in HGFs, whereas treatment with Aβ1-42 and D-galactose reduced TMEM140 expression in neuron-like cells. When exposed to the same batch of conditioned medium, neuron-like cells with TMEM140 knockdown displayed more evident injury and senescence-related phenotypes, including reduced cell viability, increased reactive oxygen species (ROS) production, a higher percentage of senescence-associated β-galactosidase (SA-β-Gal)-positive cells, and marked upregulation of IL-1β, IL-6, TNF-α, p16, p21, RELA, NFKBIA, and TP53, indicating that reduced TMEM140 expression may contribute to enhanced susceptibility of neuron-like cells to inflammatory stress.

**Conclusion:**

Through integrated transcriptomic analysis together with *in vitro* experimental validation, this study indicates that TMEM140 may be a candidate bridge molecule connecting PD and AD comorbidity. TMEM140 may participate in shaping the peripheral-central immunosenescence network and contribute to the cross-system transmission of inflammatory signaling.

## Introduction

Periodontitis (PD) is a prevalent chronic inflammatory disorder among middle-aged and elderly individuals. Under the 2017 PD staging and grading classification, moderate-to-severe PD (Stage II-III/IV) is frequently associated with sustained inflammatory burden and may substantially affect overall systemic health (Bertolini and Clark, 2024; Papapanou et al., 2018). Evidence indicates that oral microbial dysbiosis and chronic systemic inflammatory burden are strongly linked to an elevated risk of Alzheimer’s disease (AD) (Borsa et al., 2021; Ide et al., 2016; Villar et al., 2024). Animal and histological studies have shown that periodontal pathogens and their virulence factors, including *Porphyromonas gingivalis* and its gingipains, are detectable in the brain tissues of patients with AD and can trigger Aβ deposition and neuroinflammation, implying that PD may influence brain function through an “oral infection → peripheral inflammation → central pathology” cascade (Chuang et al., 2024; Dominy et al., 2019; Yoshida et al., 2023). Moreover, secreted products from *P. gingivalis*, such as outer membrane vesicles (OMVs), may traverse the blood-brain barrier or activate brain-resident immune cells through the circulation, further exacerbating neuroinflammation (Chuang et al., 2024; Walker et al., 2023; Yoshida et al., 2023).

The “peripheral inflammation-central immunity-brain aging” axis (peripheral→central immune→brain aging) has been regarded as an important mechanism underlying the association between PD and AD (Liu et al., 2024). Chronic peripheral inflammation may activate microglia and astrocytes via signaling mediators including circulating cytokines, invasive microbes, and microvesicles, leading to neuroinflammation and worsening Aβ/tau pathology as well as cognitive dysfunction. These effects are especially pronounced in the setting of inflammaging (Kosyreva et al., 2022; Li et al., 2015; Zhang et al., 2023). However, systematic studies combining transcriptomic analysis with *in vitro* functional validation remain limited, and the specific aging-related molecules connecting these two disorders have yet to be clearly defined. Moreover, accumulating evidence suggests that molecules involved in immune regulation, extracellular matrix remodeling, and metabolic homeostasis, such as TIMP1 and ALDH2, may contribute to disease progression in both PD and AD (Chen et al., 2017; Fan et al., 2023; Gui et al., 2016; Nonaka et al., 2024; Saha et al., 2020; Wang et al., 2024). These observations point to a complex network shaped by the interplay of immune, matrix, and metabolic pathways, which may offer novel targets for future combinatorial intervention strategies. From a methodological perspective, current studies generally use standardized workflows, including differential expression analysis, batch-effect correction (e.g., limma and ComBat/sva), WGCNA-based co-expression networks, and CIBERSORT immune deconvolution, to enhance the reliability and reproducibility of the data (Langfelder and Horvath, 2008; Leek et al., 2012; Newman et al., 2015; Ritchie et al., 2015).

Through integrated analysis of publicly available transcriptomic datasets and machine learning-based prioritization of candidate genes, this study identified aging-related genes shared between PD and AD and further selected TMEM140 as a major candidate molecule for detailed evaluation. The findings demonstrated that TMEM140 exhibited an upward trend in PD but a downward trend in AD, and was strongly correlated with patterns of immune cell infiltration. Additional *in vitro* experiments indicated that, when exposed to the same batch of PD-derived conditioned medium, SH-SY5Y neuron-like cells with TMEM140 knockdown exhibited exacerbated oxidative stress, inflammatory responses, and cellular senescence-associated phenotypes, implying that TMEM140 may be involved in peripheral inflammation-driven brain aging and may exert a modulatory role in this process.

## Materials and methods

### Data collection

A total of three PD datasets (GSE10334, GSE223924, and GSE16134) and three AD datasets (GSE122063, GSE132903, and GSE110226) were obtained from the NCBI GEO database. Table 1 summarizes the details of these datasets, including the microarray platforms, sample groups, and sample numbers.


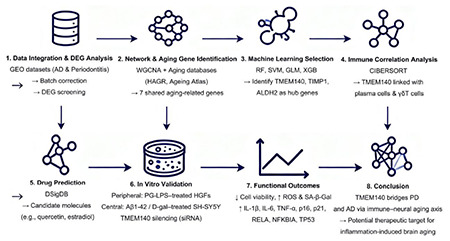


**TABLE 1:** Basic information of GEO datasets used in the study.

ID	GSE series	Disease	Samples	Platform	Group
1	GSE10334	Periodontitis	182 Periodontitis patients and 64 normal controls	GPL570	Discovery cohort
2	GSE223924	Periodontitis	20 Periodontitis patients and 10 normal controls	GPL24676	Discovery cohort
3	GSE16134	Periodontitis	241 Periodontitis patients and 69 normal controls	GPL570	Validation cohort
4	GSE122063	AD	56 AD patients and 44 normal controls	GPL16699	Discovery cohort
5	GSE132903	AD	102 AD patients and 81 normal controls	GPL10558	Discovery cohort
6	GSE110226	AD	7 AD patients and 6 normal controls	GPL10379	Validation cohort

### Data preprocessing

The two PD discovery datasets (GSE10334 and GSE223924) and the two AD discovery datasets (GSE122063 and GSE132903) were first combined separately; probe IDs were then mapped to gene symbols according to the official annotation files for each microarray platform, expression values for multiple probes corresponding to the same gene were averaged, probes lacking gene symbol annotation were excluded, and batch effects among datasets were further adjusted using the SVA package.

### Identification of DEGs

Differentially expressed genes (DEGs) in the PD and AD datasets were identified independently using the limma package in R, with |log2 fold change (FC)| > 0.25 and *P* < 0.05 as the initial criteria, while the false discovery rate (FDR)-adjusted *P*-value (adj. *P* < 0.05), calculated using the Benjamini-Hochberg procedure, was applied as the statistical threshold to rigorously control false positives; the selected results were visualized as volcano plots in R (version 4.4.2).

### Weighted gene co-expression network analysis (WGCNA)

The R package “WGCNA” was used to construct a weighted gene co-expression network to identify biologically meaningful co-expression modules and to investigate the relationships between gene networks and disease phenotypes. First, the expression matrix underwent quality control and outlier samples were excluded, after which the top 10,000 most variable genes were selected for subsequent analyses to minimize noise and enhance computational efficiency. Next, the optimal soft-thresholding power was selected within a β range of 1–20 using the pickSoftThreshold function, and a signed network was constructed to ensure approximate scale-free topology (scale-free topology fit index, *R*^2^≥ 0.85), thereby supporting the stability and biological validity of the network. The adjacency matrix was subsequently converted into a topological overlap matrix (TOM), and gene dissimilarity (1-TOM) was calculated for clustering analysis. Genes were clustered using average linkage hierarchical clustering, and the initial co-expression modules were identified by applying the dynamic tree cut algorithm. The minimum number of genes per module (minModuleSize) was set at 50, and the merging threshold for similar modules (MEDissThres) was set at 0.25 to preserve module consistency and avoid over-fragmentation or over-merging. Finally, the correlations between module eigengenes (MEs) and disease phenotypes were calculated, yielding Spearman correlation coefficients and their corresponding *P*-values. In addition, module membership (MM) and gene significance (GS) were calculated for each gene to screen for hub genes showing the strongest associations with disease.

### Identification of aging-associated genes and functional enrichment analysis

In total, 1,037 aging-related genes were collected from the Human aging Genomic Resources (HAGR), the aging Atlas, and published studies. To minimize the risk of omitting important genes, the DEGs and key module genes from each disease were merged separately after removing duplicate genes. These gene sets were then intersected with the aging-related gene list for both diseases, and the overlaps were visualized using Venn diagrams. Gene Ontology (GO) enrichment analysis, covering biological processes, cellular components, and molecular functions, was conducted using the clusterProfiler package.

### Machine learning

To accurately screen core targets, a feature set was established using the seven hub genes identified above, and four machine learning algorithms—support vector machine (SVM), random forest (RF), generalized linear model (GLM), and XGBoost (XGB) (Biecek, 2018)—were applied to identify candidate biomarker genes for PD and AD; a nomogram based on the best-performing model was subsequently constructed to assess diagnostic utility. All models underwent a unified preprocessing procedure: expression values of the seven candidate genes were standardized using Z-scores prior to feature input to remove scale differences, and a global random seed (seed = 123) was set to ensure reproducibility; hyperparameters were optimized through five-fold grid search (RF: ntree = 500, mtry = 3; SVM: radial basis function kernel, cost = 1, gamma = 0.1; GLM: binomial distribution with a logit link; XGB: max_depth = 3, learning_rate = 0.1, n_estimators = 100), and nested five-fold cross-validation was performed to reduce overfitting risk, with the inner loop used for hyperparameter tuning and the outer loop for evaluating model generalizability. To comprehensively assess model performance, the DALEX package was used to visualize the residual distributions of the four algorithms, and root mean square error (RMSE) together with the area under the receiver operating characteristic curve (AUC) was used for integrated evaluation, prioritizing the model with the lowest RMSE and highest AUC; XGBoost was ultimately selected as the optimal model, and the three key genes identified by this model were considered core candidate biomarkers for PD- and AD-related phenotypes. In the model validation stage, ROC curves were generated and AUC values were calculated using the “pROC” package in R to quantify predictive performance, and the model’s external applicability was further evaluated using an independent validation dataset to further rule out overfitting.

### Construction and validation of the nomogram

A nomogram model was constructed based on the candidate core genes using the “rms” package in R. In the nomogram, “Points” indicate the score assigned to each candidate gene, whereas “Total Points” indicate the sum of the scores for all included genes. Furthermore, the predictive performance of the nomogram was assessed using calibration curves and decision curve analysis (DCA). In the AD and PD datasets, *t*-tests of the core genes were conducted separately using R packages including limma and ggplot2, and boxplots were drawn to illustrate differences in gene expression across groups. Finally, the predictive accuracy of the core genes was assessed by ROC curve analysis.

### Immune cell infiltration and correlation analyses

In this study, the CIBERSORT algorithm (with default parameters and the LM22 signature matrix) was applied to evaluate the relative proportions of 22 immune cell subtypes in control and disease samples from the AD and PD datasets (Newman et al., 2019). The input matrix strictly fulfilled the requirements for CIBERSORT analysis, consisting of a normalized and batch-corrected gene expression matrix, with only genes showing expression levels > 1 in at least 20% of samples retained to ensure compliance with CIBERSORT quality control criteria for expression data. In the CIBERSORT analysis, *P*-values for immune cell proportions were calculated based on 1,000 permutations, and multiple testing correction for all cell types was performed using the Benjamini-Hochberg method; only results with adjusted *P* < 0.05 were included in subsequent analyses. Based on the quality-controlled immune cell abundance data, Spearman’s rank correlation analysis (two-sided test) was used to assess the association between TMEM140 expression levels and the relative abundance of immune cells, and the resulting correlations were further subjected to FDR correction, with adjusted *P* < 0.05 considered statistically significant, to control false positives arising from multiple comparisons.

### Single-cell RNA sequencing analysis

For the single-cell RNA sequencing (scRNA-seq) analysis, AD-related single-cell data were retrieved from the Gene Expression Omnibus (GEO) database (GSE157827). This study included samples from two healthy controls and two patients with AD in this dataset, all of which were derived from the prefrontal cortex. To ensure data reliability, stringent quality control criteria were adopted, retaining cells with a mitochondrial gene proportion < 20%, 200–5,000 expressed genes per cell with at least 200 detected genes, and genes expressed in at least three cells. To correct batch effects and improve clustering accuracy, data integration was performed using the Harmony algorithm; following log normalization, the top 2,000 highly variable genes were identified with the FindVariableFeatures function. Subsequently, principal component analysis (PCA) was used for dimensionality reduction, followed by soft k-means clustering integrated with the Harmony package, and cells were classified into distinct clusters using the FindClusters function with a resolution of 0.4. Cell types were annotated based on known marker genes and visualized with an annotation heatmap; the annotated cell populations and their distributions were then displayed using the t-SNE algorithm, and the expression patterns of core target genes across different cell types were further investigated.

### Screening of candidate drugs

To identify candidate drugs that may target the shared pathological mechanisms of AD and PD, this study performed an analysis using the Drug Signature Database (DSigDB) through the Enrichr online platform.

### PD model construction and treatment of human gingival fibroblasts

#### Culture of human gingival fibroblasts (HGFs)

HGFs were isolated *in vitro* and maintained in DMEM/F12 medium (Servicebio, G4612-500ML) containing 10% fetal bovine serum (FBS; Gibco, A5669701) and 1% penicillin/streptomycin (Biosharp, BL142A). Cells were cultured routinely in a constant-temperature incubator at 37°C with 5% CO_2_.

#### Concentration screening of *Porphyromonas gingivalis* lipopolysaccharide (PG-LPS)

HGFs at the logarithmic growth phase were plated at an appropriate density and maintained under standard culture conditions. To identify the optimal inflammatory induction conditions, HGFs were exposed to *Porphyromonas gingivalis* lipopolysaccharide (PG-LPS; InvivoGen, tlrl-ppglps; derived from *P. gingivalis* ATCC 33277) at final concentrations of 0.1, 0.5, 1, 5, and 10 μg/mL for 24 h. After treatment, the culture supernatant was removed, and CCK-8 reagent (Biosharp, BL1055C) was added and incubated at 37°C for 2 h. The optical density (OD) was subsequently measured at 490 nm using a microplate reader (Varioskan LUX, Thermo) to evaluate cell viability.

#### Conditioned medium (CM) preparation and standardization

To mimic the chronic inflammatory microenvironment related to PD, P3 HGFs were seeded at 5 × 10^4^ cells/cm^2^ and grown to approximately 80% confluence, and then stimulated with 1 μg/mL PG-LPS for 24 h under strictly standardized culture conditions. All CM samples were generated from the same cell batch and prepared using the same passage, seeding density, and stimulation conditions. After stimulation, the culture supernatant was collected, centrifuged at 1,000 x g for 10 min to remove cellular debris, and filtered through a 0.22 μm membrane to obtain CM; the CM was then applied to SH-SY5Y cells at volume ratios of 0, 25, 50, and 100% for 48 h, and cell viability was evaluated using the CCK-8 assay to identify the optimal treatment concentration, which was consistently used in subsequent experiments to standardize the intervention conditions. Furthermore, ELISA was used to quantitatively determine IL-6, IL-1β, and TNF-α levels in the CM to confirm consistent exposure to key inflammatory mediators, thereby enhancing the reliability and reproducibility of the experimental findings.

### Construction of the neuron-like cell model

#### Culture of the human neuroblastoma cell line SH-SY5Y

SH-SY5Y human neuroblastoma cells (Procell, CL-0208) were maintained in DMEM/F12 containing 10% FBS and 1% penicillin/streptomycin. Cells were maintained under standard culture conditions at 37°C in a humidified incubator with 5% CO_2_.

#### Construction of the cellular senescence model

Log-phase SH-SY5Y cells were exposed to 250 μM D-galactose (Solarbio, SG8010) for 24 h to establish a cellular senescence model.

#### Construction of the AD model

Log-phase SH-SY5Y cells were exposed to 100 μM Aβ1-42 (RoyoBiotech) for 24 h to establish an AD-like model.

#### Assessment of residual LPS effects

SH-SY5Y cells were subjected to the following treatments for 48 h:

Control+SH-SY5Y: treated with standard culture medium.

HGFs+SH-SY5Y: treated with normal HGFs culture supernatant without LPS stimulation.

PG-LPS+SH-SY5Y: treated with medium directly supplemented with LPS at the same concentration used during CM preparation.

CM+SH-SY5Y: treated with standardized conditioned medium collected from LPS-stimulated HGFs.

Following 48 h of treatment, cell viability was evaluated by the CCK-8 assay to determine whether the biological effects of CM were attributable to residual LPS.

#### siRNA transfection

siRNA transfection was carried out in 24-well plates when SH-SY5Y cells reached approximately 70% confluence, with about 2 × 10^5^ cells seeded per well. After mixing 1.25 μL of 20 μM TMEM140 siRNA stock with 30 μL of 1 × riboFECT™ CP Buffer, 3 μL of riboFECT™ CP Reagent was added, gently mixed by pipetting, and incubated at room temperature for 15 min to allow formation of transfection complexes. The transfection complexes were subsequently added dropwise to wells containing antibiotic-free complete medium, gently mixed, and cultured at 37°C in 5% CO_2_ for 48 h.

The experimental groups were as follows:

①: Con-SY5Y: control group without gene knockdown.

②: siTMEM140-SY5Y: TMEM140-silenced group.

After 48 h of transfection, efficiency was assessed using an inverted fluorescence microscope (Nikon, CSIM100), and a fluorescence-positive rate of ≥ 70% was regarded as successful transfection.

#### Western blot analysis

Total protein was extracted from Con-SY5Y and siTMEM140-SY5Y cells using RIPA lysis buffer (Thermo, 89901), and protein concentration was measured with a BCA assay kit (Thermo, 23235). Equal amounts of protein were separated by SDS-PAGE, transferred onto PVDF membranes, and blocked with 5% skim milk for 1 h at room temperature. The membranes were subsequently incubated overnight at 4°C with primary antibodies against TMEM140 (Invitrogen, PA5-144108) and GAPDH (Invitrogen, MA5-15738), washed, and then incubated with HRP-labeled secondary antibodies for 1 h at room temperature. Signals were developed using ECL chemiluminescence, and band intensity was quantified by grayscale analysis with ImageJ software.

#### Conditioned medium treatment

SH-SY5Y cells were treated for 48 h with the same batch of standardized CM and assigned to the following groups:

①:CM+SH-SY5Y.

②:CM+siNC+SH-SY5Y.

③:CM+siTMEM140+SH-SY5Y.

In these groups, siNC was used as the negative transfection control, whereas siTMEM140 was used to silence TMEM140 expression.

#### CCK-8 assay for cell viability

After CM treatment, the culture supernatant was removed from SH-SY5Y cells, the cells were washed once with PBS, and CCK-8 working solution was added followed by incubation at 37°C for 2 h. Optical density was subsequently measured at 490 nm with a microplate reader to assess cell viability.

#### Intracellular reactive oxygen species (ROS) assay

Intracellular ROS levels were measured using the DCFH-DA fluorescent probe method. Following CM treatment, the cells were washed once with PBS, and 200 μL of 10 μmol/L DCFH-DA working solution (Solarbio, ID35609) was added to each well and incubated at 37°C for 30 min. After washing, the fluorescence intensity was observed and recorded using a fluorescence microscope.

#### Senescence-associated β-galactosidase (SA-β-Gal) staining

After CM treatment, the culture supernatant was removed, the cells were washed once with PBS, and fixed with 1 mL of fixative at room temperature for 15 min. The fixative was then discarded, and the cells were washed three times with PBS for 3 min each, followed by the addition of 1 mL of SA-β-Gal staining working solution (Solarbio, BC2585); the plate was sealed and incubated overnight at 37°C. The staining results were examined under an inverted microscope on the following day.

#### Quantitative real-time PCR analysis

Total RNA was extracted from cells using TRIzol reagent. Reverse transcription was carried out using the PrimeScript RT Reagent Kit with gDNA Eraser (Takara) to eliminate genomic DNA contamination and synthesize cDNA. Quantitative real-time PCR (qRT-PCR) was performed using the QuantiNova™ SYBR Green PCR Kit (Qiagen) on a Bio-Rad CFX Connect system. Relative gene expression was calculated using the 2^∧^-ΔΔCt method.

### Statistical analysis

All experiments were conducted with at least three independent biological replicates. Data are expressed as mean ± standard deviation (Mean ± SD). All statistical analyses were carried out using SPSS 17.0 software. Differences between two groups were analyzed using the independent-samples *t*-test, while comparisons among multiple groups were conducted by one-way ANOVA, followed by *post-hoc* multiple-comparison testing when appropriate. Prior to statistical analysis, the data were tested for normality and homogeneity of variance. Differences were considered statistically significant at *P* < 0.05.

## Results

### Identification of DEGs

**FIGURE 1:**
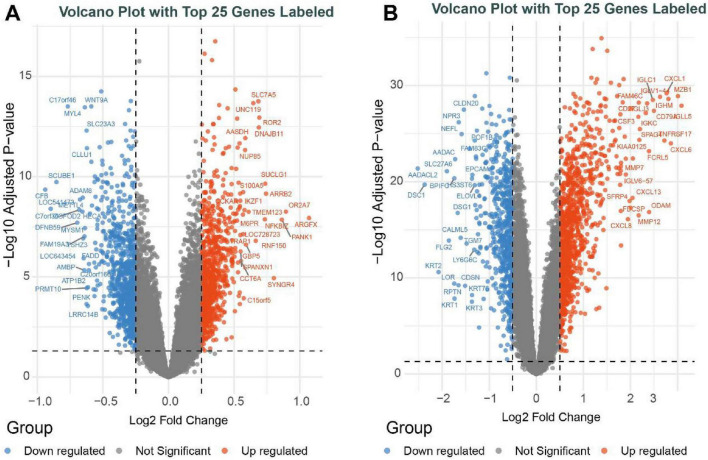
Identification of differentially expressed genes. **(A)** Volcano plot showing the DEGs between PD and healthy controls. **(B)** Volcano plot showing the DEGs between AD and healthy controls.

Raw data for the PD and control groups were retrieved from the GEO database, integrated following batch effect correction, and further normalized to construct an integrated PD training cohort consisting of 202 PD cases and 74 control samples. Likewise, the raw datasets for the AD and control groups were combined after batch effect correction, yielding an integrated AD training cohort composed of 158 AD cases and 125 control samples, and the batch effects were substantially reduced after these correction procedures. In the PD cohort, a total of 1,703 DEGs were identified, of which 1,090 were upregulated and 613 were downregulated (Figure 1A). In the AD cohort, a total of 1,755 DEGs were identified, among which 865 were upregulated and 890 were downregulated (Figure 1B).

### Weighted gene co-expression network analysis in PD and AD

**FIGURE 2:**
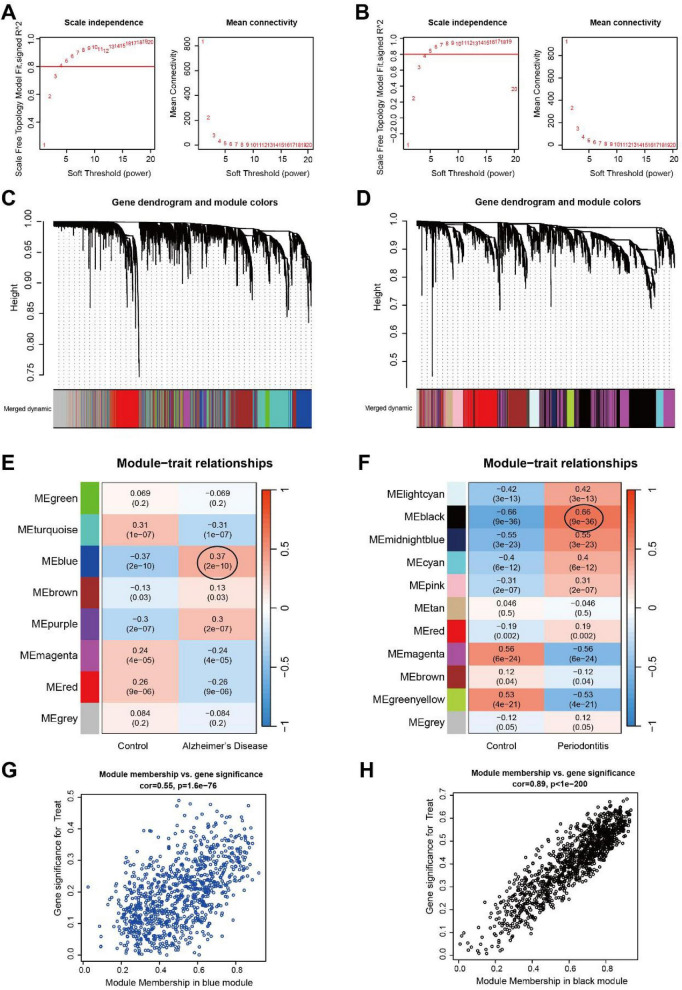
WGCNA analysis in AD and PD. **(A)** Mean connectivity and soft-thresholding power (β) for evaluating scale independence in the integrated AD cohort (GSE122063 and GSE132903). **(B)** Mean connectivity and soft-thresholding power (β) for evaluating scale independence in the integrated PD cohort (GSE10334 and GSE223924). **(C)** Gene clustering dendrogram for AD. **(D)** Gene clustering dendrogram for PD. **(E)** Heatmap showing correlations between module eigengenes and clinical phenotypes in AD (red indicates positive correlation, and blue indicates negative correlation). **(F)** Heatmap showing correlations between module eigengenes and clinical phenotypes in PD (red indicates positive correlation, and blue indicates negative correlation). **(G)** Scatter plot of gene significance (GS) versus module membership (MM) for key AD-related module genes. **(H)** Scatter plot of gene significance (GS) versus module membership (MM) for key PD-related module genes.

WGCNA was conducted separately on the integrated PD and AD datasets to investigate the associations between clinical characteristics and gene expression. The optimal soft-threshold power was set at 6 for both the AD and PD datasets (Figures 2A,B), and according to the similarity among modules, a total of 8 modules were identified in the AD dataset and 11 modules in the PD dataset (Figures 2C,D). Correlation analysis between modules and traits revealed that the blue module was most positively correlated with AD (*r* = 0.37) (Figure 2E), while the black module exhibited the strongest positive correlation with PD (*r* = 0.66) (Figure 2F). Significant correlations were also observed between gene significance (GS) and module membership (MM) within these modules (correlation coefficient = 0.55 for AD and 0.89 for PD) (Figures 2G, H), further supporting that the genes in these modules were strongly associated with disease development and might contribute to the pathological progression of AD and PD.

### Identification of senescence-related genes and functional enrichment analysis

**FIGURE 3:**
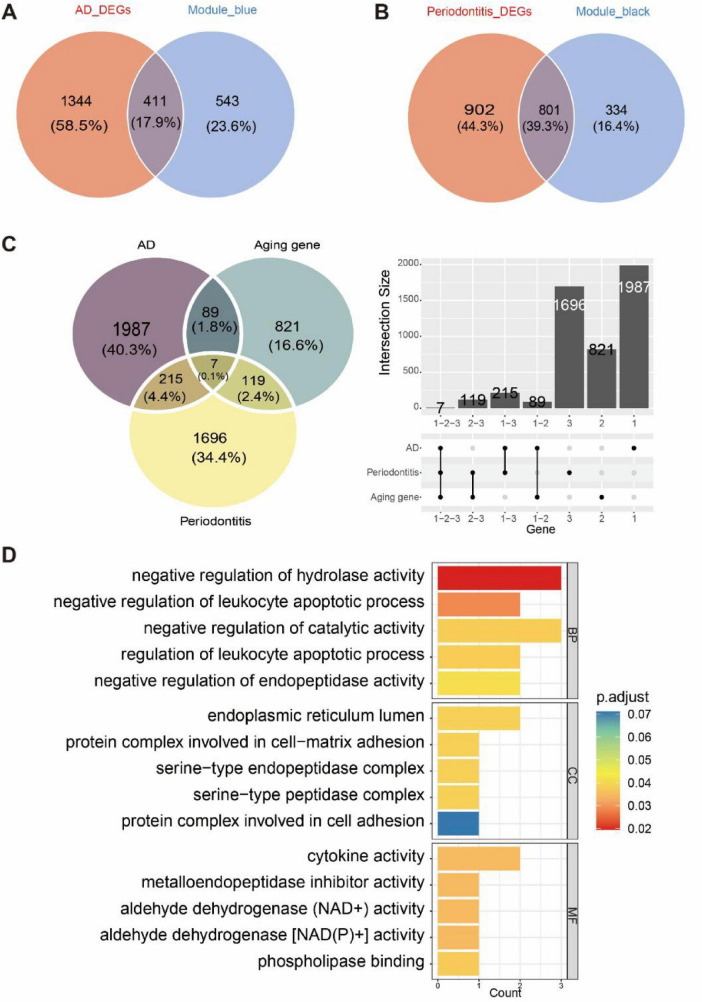
Identification of shared aging-related genes in AD and PD. **(A)** Union set of AD DEGs and genes in the blue module. **(B)** Union set of PD DEGs and genes in the black module. **(C)** Venn diagram of the overlap between AD/PD shared genes and aging-related genes. **(D)** GO enrichment analysis of the shared aging-related genes in AD and PD.

By taking the union of AD DEGs and blue module genes, we obtained 2,298 genes (Figure 3A), while the union of PD DEGs and black module genes yielded 2,037 genes (Figure 3B); intersection analysis between the aging-related gene set and the merged gene sets from the two diseases ultimately identified seven aging-related genes shared by AD and PD (Figure 3C), indicating that these genes may be commonly involved in the pathogenesis of both disorders and provide candidate targets for subsequent key gene identification. Based on these findings, GO enrichment analysis was conducted for the seven shared aging-related genes to define their overall functional characteristics and the biological pathways commonly involved, and the results indicated that, in the biological process (BP) category, the gene set was significantly enriched in immune- and cellular metabolism-related processes, including negative regulation of hydrolase activity, negative regulation of leukocyte apoptotic process, and negative regulation of catalytic activity; in the cellular component (CC) category, it was mainly enriched in structure- and adhesion-related components such as the endoplasmic reticulum lumen, protein complexes involved in cell-matrix adhesion, and serine-type endopeptidase complexes; in the molecular function (MF) category, it was significantly enriched in immune regulatory and enzyme activity-related functions, including cytokine activity, metallopeptidase inhibitor activity, aldehyde dehydrogenase (NAD+) activity, and phospholipase binding (Figure 3D). These enrichment findings reveal the overall functional landscape of the aging-related genes shared by AD and PD and provide pathway-level clues for further exploration of the potential mechanisms of individual key genes.

### Development and evaluation of machine learning models

**FIGURE 4:**
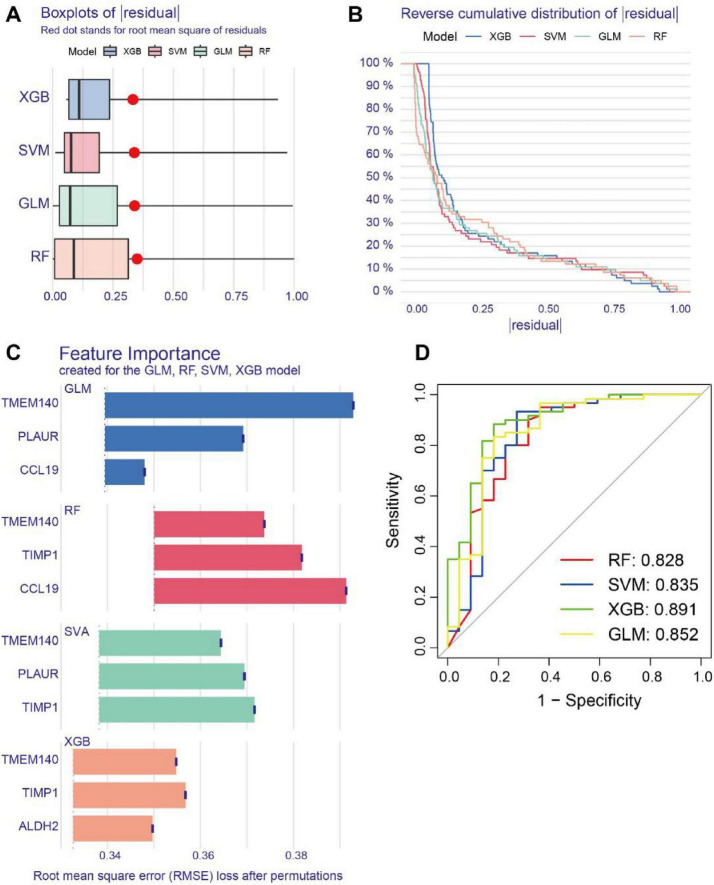
Machine learning-based identification of core genes. **(A)** Boxplot illustrating the residual distributions of the machine learning models. The root mean square error (RMSE) of the residuals is represented by red dots. **(B)** Cumulative residual distributions of the machine learning models. **(C)** Importance ranking of genes across the four machine learning models. **(D)** ROC curves for the four machine learning models.

**FIGURE 5:**
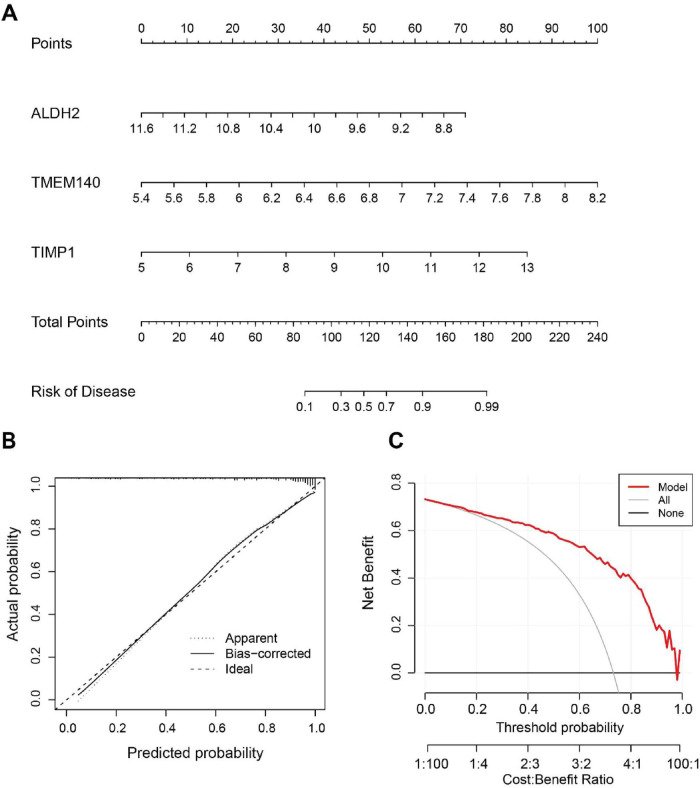
Development of the nomogram. **(A)** Nomogram constructed using the key genes ranked by importance in the XGB model. **(B)** Calibration curve analysis for evaluating the predictive accuracy of the nomogram model. **(C)** Decision curve analysis (DCA) curves for assessing the clinical utility of the nomogram model.

Using the seven shared senescence-related intersecting genes between AD and PD identified in the preliminary analyses, this study employed four machine learning models (RF, SVM, GLM, and XGB) to further identify core biomarkers with high diagnostic potential. First, the residual distributions of the four models were assessed and compared, showing that the XGB model had markedly lower residuals than the other models (Figures 4A,B); the top three genes in each model were then ranked by importance according to root mean square error (RMSE) (Figure 4C). Furthermore, receiver operating characteristic (ROC) curves generated through five-fold cross-validation were used to assess the diagnostic performance of the four machine learning algorithms in the training dataset, and all four models achieved area under the curve (AUC) values above 0.8, with the XGB model showing the highest AUC (AUC = 0.891), while the AUC values for RF, SVM, and GLM were 0.828, 0.835, and 0.852, respectively (Figure 4D). Taking both model residuals and AUC values into account, the XGB model was ultimately identified as the optimal model, and the top three genes ranked by this model among the seven shared senescence-related intersecting genes were selected as the three core genes: TMEM140, ALDH2, and TIMP1. A nomogram model was further established based on these three core genes to evaluate disease risk (Figure 5A), and calibration curve analysis demonstrated good consistency between the model’s predictions and the ideal model (Figure 5B); decision curve analysis (DCA) further showed that the model yielded greater net benefit than extreme decision strategies over a wide range of threshold probabilities, indicating that the model built from these three core genes may have potential clinical utility (Figure 5C).

### Expression and diagnostic validation of core genes in AD and PD

**FIGURE 6:**
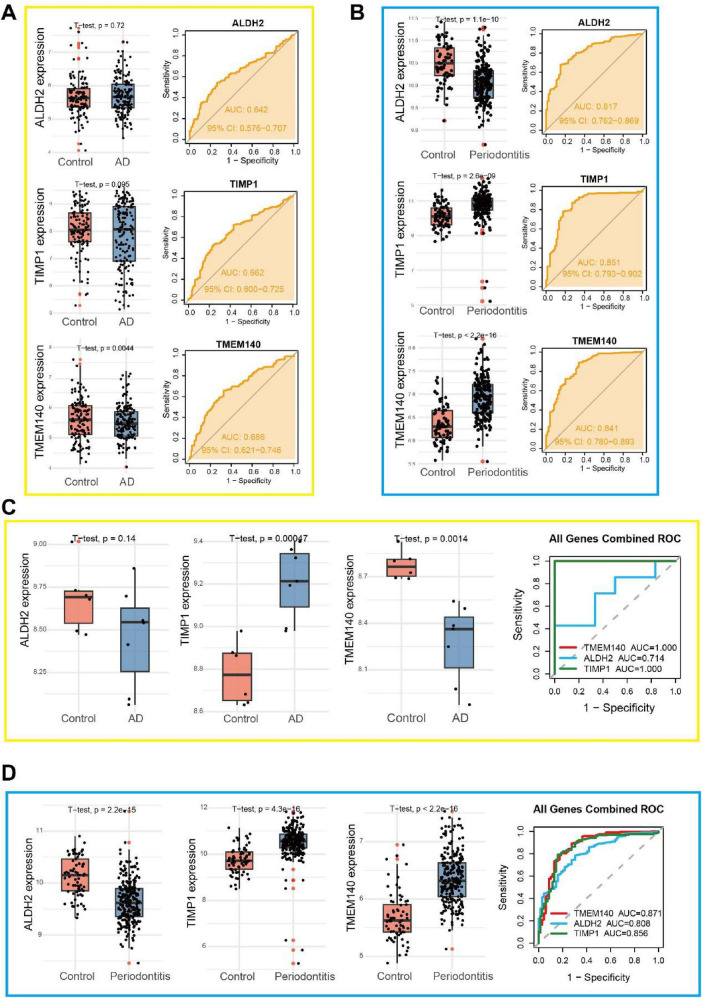
Validation of the three hub genes in AD and PD cohorts. **(A)** Expression profiles and ROC curves of the three hub genes in the integrated AD cohort (GSE122063 and GSE132903). **(B)** Expression profiles and ROC curves of the three hub genes in the integrated PD cohort (GSE10334 and GSE223924). **(C)** Expression profiles and ROC curves of the three hub genes in the AD validation cohort (GSE110226). **(D)** Expression profiles and ROC curves of the three hub genes in the PD validation cohort (GSE16134).

Independent training-set screening and validation-set testing were performed to assess the expression differences and diagnostic performance of the three hub genes in AD and PD. In the integrated AD training cohort (GSE122063 and GSE132903) (Figure 6A), TMEM140 expression was significantly reduced in the AD group compared with the control group (*P* = 0.0044), while TIMP1 and ALDH2 did not differ significantly between groups. ROC analysis revealed that the AUC values for ALDH2, TIMP1, and TMEM140 were 0.642, 0.662, and 0.686, respectively, indicating that all three genes possessed basic diagnostic value for distinguishing AD, with TMEM140 exhibiting the strongest discriminatory ability among them. In the integrated PD training cohort (GSE10334 and GSE223924) (Figure 6B), ALDH2 expression was significantly decreased in the PD group relative to controls (*P* < 0.0001), whereas TIMP1 and TMEM140 were significantly upregulated compared with controls (*P* < 0.0001). ROC analysis showed that the three genes achieved AUC values of 0.817, 0.851, and 0.841, respectively, all demonstrating favorable diagnostic performance. In the independent AD validation cohort (GSE110226; 7 AD cases and 6 controls) (Figure 6C), the expression patterns of TMEM140 and TIMP1 were consistent with those observed in the training cohort, with TMEM140 significantly downregulated in the AD group (*P* = 0.0014) and TIMP1 significantly upregulated (*P* = 0.00047), while ALDH2 remained not significantly different between groups. ROC analysis showed that both TMEM140 and TIMP1 achieved AUC values of 1.000; however, this finding may be attributable to the very small sample size of the validation cohort and likely reflects an apparently ideal discriminatory trend in a limited sample, such that their diagnostic utility for AD cannot yet be conclusively determined. In the independent PD validation cohort (GSE16134) (Figure 6D), the expression trends of all three genes were entirely consistent with those in the training cohort, and the AUC values for ALDH2, TIMP1, and TMEM140 were 0.808, 0.856, and 0.871, respectively, further validating the stable moderate-to-high diagnostic performance of TMEM140 for PD. Based on the results of training-set screening and independent validation, TMEM140 showed consistent and significant differential expression in both AD and PD, indicating that it is a shared aging-related gene between the two disorders. Specifically, in PD, this gene demonstrated stable diagnostic discrimination in both the training set and the large validation cohort, whereas in AD it showed only moderate discriminatory value in the training set, and the ideal performance observed in the small validation cohort still requires confirmation in larger independent cohorts. Overall, TMEM140 appears to be a core aging-related gene underlying PD-AD comorbidity and warrants further in-depth study.

### Analysis of immune cell infiltration

**FIGURE 7:**
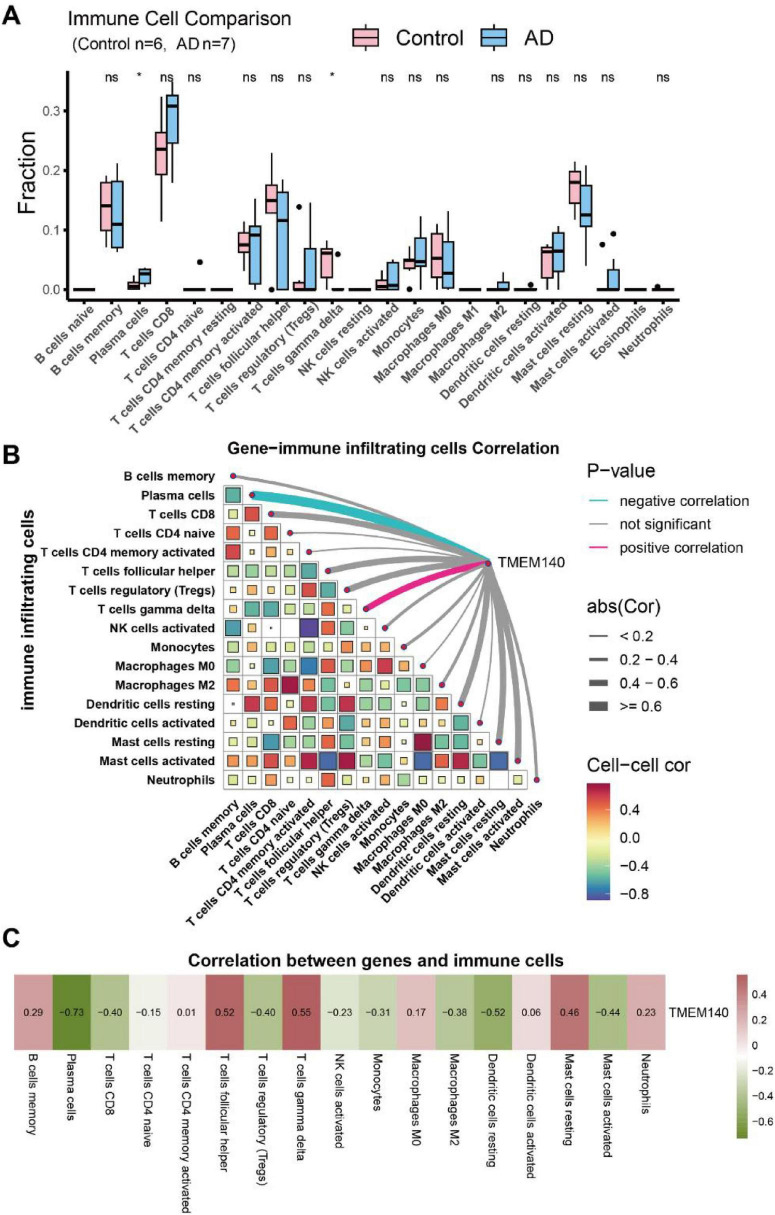
Analysis of immune cell infiltration in AD. **(A)** Boxplot showing immune cell infiltration patterns in the AD dataset (GSE110226) (*P < 0.05). **(B)** Association between TMEM140 and immune cells. **(C)** Heatmap showing the correlations between TMEM140 and immune cells.

**FIGURE 8:**
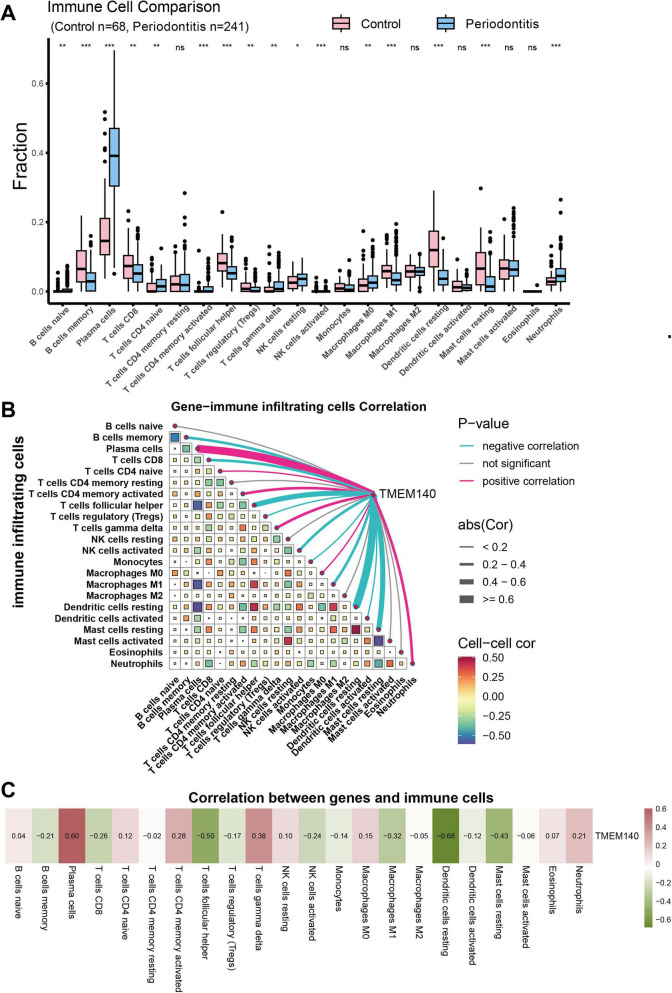
Analysis of immune cell infiltration in PD. **(A)** Boxplot showing immune cell infiltration patterns in the PD dataset (GSE16134) (*P < 0.05; **P < 0.01; ***P < 0.001). **(B)** Association between TMEM140 and immune cells. **(C)** Heatmap showing the correlations between TMEM140 and immune cells.

The relative proportions of 22 immune cell types in each sample were estimated using the CIBERSORT algorithm. The results indicated that in AD (Figure 7A), relative to normal samples, AD samples exhibited significantly increased plasma cell infiltration and significantly decreased γδ T-cell infiltration. Further correlation analysis showed (Figures 7B,C) that TMEM140 was most negatively correlated with plasma cells and most positively correlated with γδ T cells, indicating that TMEM140 expression is closely linked to the infiltration levels of plasma cells and γδ T cells and is associated with the immune microenvironment of AD. In PD (Figure 8A), immune infiltration displayed a more complex pattern: relative to normal samples, PD samples showed significantly increased infiltration of naïve B cells, plasma cells, naïve CD4^+^ T cells, activated memory CD4^+^ T cells, γδ T cells, resting NK cells, M0 macrophages, and neutrophils, whereas the infiltration of memory B cells, naïve CD8^+^ T cells, follicular helper T cells, regulatory T cells, activated NK cells, M1 macrophages, resting dendritic cells, and resting mast cells was significantly reduced. Correlation analysis demonstrated (Figures 8B,C) that TMEM140 was most negatively correlated with resting dendritic cells and most positively correlated with plasma cells, suggesting that its expression is associated with the infiltration levels of these two immune cell populations and with the immunopathological characteristics of PD. Taken together, as an aging-related gene common to AD and PD comorbidity, TMEM140 was significantly associated with plasma cell infiltration in both diseases (negatively in AD but positively in PD), and additionally showed specific associations with γδ T-cell infiltration in AD and resting dendritic cell infiltration in PD, indicating that TMEM140 may represent a key molecule closely linked to immune microenvironmental dysregulation in both disorders.

### Single-cell RNA sequencing analysis verifies the cellular expression pattern of TMEM140

**FIGURE 9:**
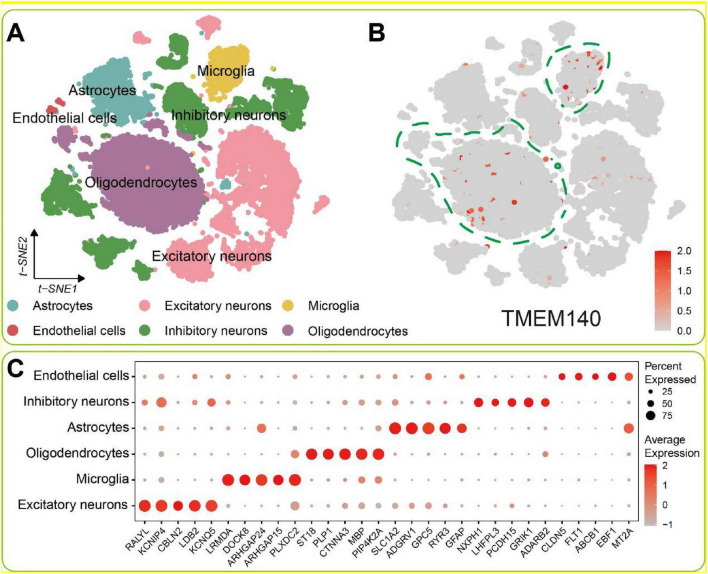
Expression profile of TMEM140 in AD single-cell data. **(A)** t-SNE-based visualization of the clustering of six major brain cell subtypes identified in the AD single-cell dataset. **(B)** t-SNE plot illustrating the cell type-specific expression distribution of TMEM140, where red indicates a higher relative expression level. **(C)** Bubble plot of representative marker genes for the six cell types, illustrating the characteristic gene expression patterns across different cell populations.

To further characterize the cell type-specific expression of TMEM140 in the central nervous system, publicly available single-cell RNA-seq data were analyzed. First, the raw data underwent quality control and normalization, after which t-SNE-based dimensionality reduction and clustering classified the cells into six major brain cell subtypes: excitatory neurons (RALY, KCNIP4, CBLN2, LDB2, KCNQ5), inhibitory neurons (NXPH1, LHFPL3, PCDH15, GRIK1, ADARB2), oligodendrocytes (ST18, PLP1, CTNNA3, MBP, PIP4K2A), astrocytes (SLC1A2, ADGRV1, GPC5, RYR3, GFAP), microglia (LRMDA, DOCK8, ARHGAP24, ARHGAP15, PLXDC2), and endothelial cells (CLDN5, FLT1, ABCB1, EBF1, MT2A) (Figures 9A,C). The results indicated that TMEM140 displayed pronounced cell type-specific expression in the brain, with predominant enrichment in glial subpopulations, particularly oligodendrocytes and microglia (Figure 9B). This expression pattern was highly consistent with the earlier findings of the present study: in the AD-like neuron model, reduced TMEM140 expression was associated with greater neuronal vulnerability to inflammatory injury and aggravated senescence-related phenotypes. At the same time, under PD-related conditions, peripheral inflammation may influence glial cells through the immune-neural axis, thereby modulating central inflammatory status and neuronal resilience. Taken together, the single-cell analysis confirmed the glial cell-specific expression of TMEM140 in the central nervous system and provided cellular-level evidence supporting its key regulatory role in peripheral inflammation-driven brain aging.

### Screening of candidate drugs

The model genes were analyzed through the Drug Signature Database (DSigDB) available on the Enrichr platform to identify potential targeted therapeutic agents. The top 10 candidate compounds were suloctidil (HL60 cell line, expression upregulated), metronidazole (PC3 cell line, expression downregulated), arsenite (CTD database ID: 00000779), disodium selenite (CTD database ID: 00007229), pirinixic acid (CTD database ID: 00000280), hydrogen peroxide (CTD database ID: 00006118), gallium chloride (CAS No. 7646-79-9; CTD database ID: 00000928), quercetin (CTD database ID: 00006679), tetradioxin (CTD database ID: 00006848), and estradiol (CTD database ID: 00005920) (Table 2).

**TABLE 2:** Candidate drugs targeting AD- and PD-related genes.

Term	*P*-value	Combined score	Genes
Suloctidil HL60 UP	0.007049917	98396.16911	TMEM140
Metronidazole PC3 DOWN	0.037249808	63351.03909	TMEM140
Arsenite CTD 00000779	0.064999768	51114.04839	TMEM140
Disodium selenite CTD 00007229	0.068499765	49945.63202	TMEM140
Pirinixic acid CTD 00000280	0.070099764	49430.4312	TMEM140
Hydrogen peroxide CTD 00006118	0.133649742	34871.15527	TMEM140
7646-79-9 CTD 00000928	0.154699743	31551.14679	TMEM140
Quercetin CTD 00006679	0.157899743	31086.87909	TMEM140
Tetradioxin CTD 00006848	0.18839975	27094.27983	TMEM140
Estradiol CTD 00005920	0.216799758	23946.82753	TMEM140

### PG-LPS-induced inflammatory injury model in HGFs and differential expression of TMEM140 in peripheral-central system-related models

**FIGURE 10:**
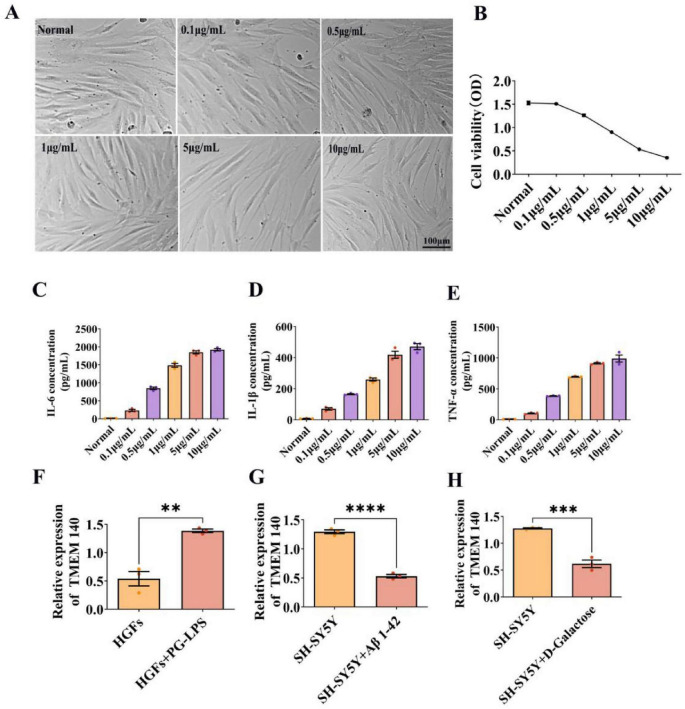
Effects of different concentrations of PG-LPS on cell viability and TMEM140 expression in HGFs. **(A)** Bright-field images of HGFs treated with different concentrations of PG-LPS (0.1, 0.5, 1, 5, and 10 μg/mL) for 24 h. **(B)** CCK-8 assay of HGFs cell viability after exposure to different concentrations of PG-LPS. **(C–E)** ELISA measurements of IL-6, IL-1β, and TNF-α secretion levels in the supernatant of HGFs following treatment with different concentrations of PG-LPS. **(F)** qRT-PCR analysis of TMEM140 expression changes in HGFs after PG-LPS treatment. **(G)** qRT-PCR analysis of TMEM140 expression changes in SH-SY5Y cells after Aβ1-42 treatment. **(H)** qRT-PCR analysis of TMEM140 expression changes in SH-SY5Y cells after D-gal treatment. Scale bar = 100 μm. “*” indicates statistical significance versus the control group (**P < 0.01, ***P < 0.001, ****P < 0.0001).

To establish a stable and physiologically relevant PG-LPS-induced inflammatory injury model in HGFs, this study exposed HGFs to different concentrations of PG-LPS (0.1, 0.5, 1, 5, and 10 μg/mL) for 24 h, and assessed cellular status through morphological observation and CCK-8 analysis. The results demonstrated that, as the concentration of PG-LPS increased, HGFs gradually exhibited morphological alterations and a clear decline in cell viability, suggesting that PG-LPS causes dose-dependent damage to HGFs (Figures 10A,B). ELISA analysis further demonstrated that the levels of IL-6, IL-1β, and TNF-α in the supernatant of HGFs progressively increased with rising PG-LPS concentrations, indicating effective activation of the inflammatory response (Figures 10C–E). Considering both the alterations in cell viability and the induction efficiency of inflammatory cytokines, 1 μg/mL PG-LPS was ultimately chosen as the optimal modeling condition for subsequent experiments. Subsequent examination of TMEM140 expression showed that, relative to the normal control group, PG-LPS treatment significantly increased TMEM140 expression in HGFs. By contrast, in central nervous system-related models, TMEM140 expression was significantly reduced in both Aβ1-42-treated SH-SY5Y cells and the D-gal-induced senescence model (Figures 10F–H). These findings suggest that TMEM140 displays opposite expression patterns in the peripheral inflammatory microenvironment and in central neurodegenerative alterations, and may participate in the coordinated regulation linking peripheral and central pathological processes.

### Establishment of standardized conditioned medium (CM) and its impact on SH-SY5Y cell viability

**FIGURE 11:**
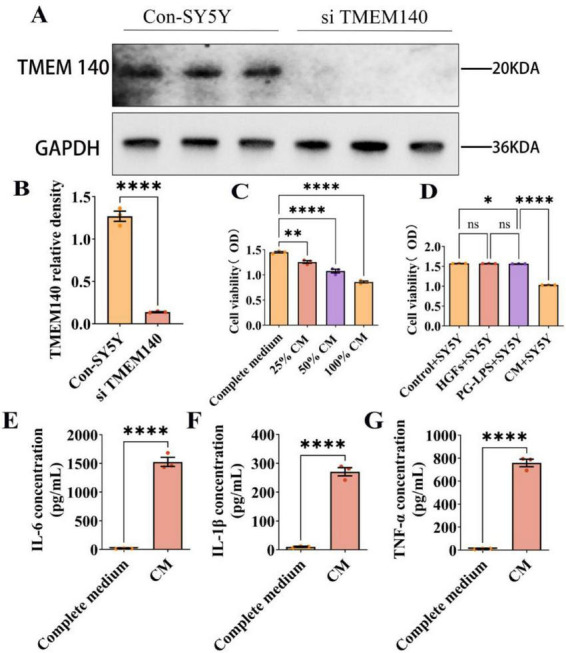
Preparation and quality evaluation of standardized CM. **(A)** Western blot results showing TMEM140 protein expression in the Con-SY5Y and siTMEM140 groups, with GAPDH as the internal reference. **(B)** Quantification of the relative band intensity of TMEM140 protein. **(C)** CCK-8 assay results of SH-SY5Y cells after 48 h treatment with standardized CM (generated from HGFs of the same batch, passage, and seeding density following 24 h stimulation with 1 μg/mL PG-LPS) diluted to 25, 50, and 100%. **(D)** Comparison of cell viability among the Control+SH-SY5Y, HGFs+SH-SY5Y, PG-LPS+SH-SY5Y, and CM+SH-SY5Y groups after 48 h treatment to assess the influence of residual LPS on the biological effects of CM. **(E–G)** ELISA measurement of the changes in IL-6, IL-1β, and TNF-α concentrations in complete medium and standardized CM. *P < 0.05, **P < 0.01, ****P < 0.0001; ns indicates that the difference is not statistically significant.

To simulate the chronic inflammatory microenvironment related to PD and establish a standardized treatment system, P3 HGFs from the same batch were seeded at 5 × 10^4^ cells/cm^2^ and grown to approximately 80% confluence before being stimulated with 1 μg/mL PG-LPS for 24 h. All CM samples were generated from HGFs of the same batch and passage under identical seeding density and stimulation conditions, and standardized CM was obtained after centrifugation of the collected supernatant at 1,000 x g and filtration through a 0.22 μm membrane. TMEM140 siRNA transfection was first performed in SH-SY5Y cells, and Western blot analysis demonstrated a significant reduction in TMEM140 expression in the siTMEM140 group, confirming successful knockdown (Figures 11A,B). SH-SY5Y cells were subsequently exposed to CM at proportions of 25, 50, and 100% for 48 h, and CCK-8 results showed a dose-dependent decline in cell viability with increasing CM concentration (Figure 11C). Residual LPS validation demonstrated no significant differences among the Control, HGFs, and PG-LPS groups, whereas the CM group exhibited significantly decreased cell viability (Figure 11D), indicating that its effect was primarily mediated by inflammation-associated secretory factors. ELISA analysis revealed that IL-6, IL-1β, and TNF-α levels in the CM were markedly increased, rising from 12.0 ± 4.6, 10.3 ± 3.1, and 11.0 ± 4.4 pg/mL to 1526.0 ± 136.6, 270.7 ± 25.1, and 758.3 ± 56.7 pg/mL, respectively (Figures 11E–G). Taken together, the standardized CM established with 1 μg/mL PG-LPS stably enriched pro-inflammatory mediators and is suitable for research on the peripheral inflammatory microenvironment.

### TMEM140 expression modulates neuronal sensitivity to PD-derived conditioned medium

**FIGURE 12:**
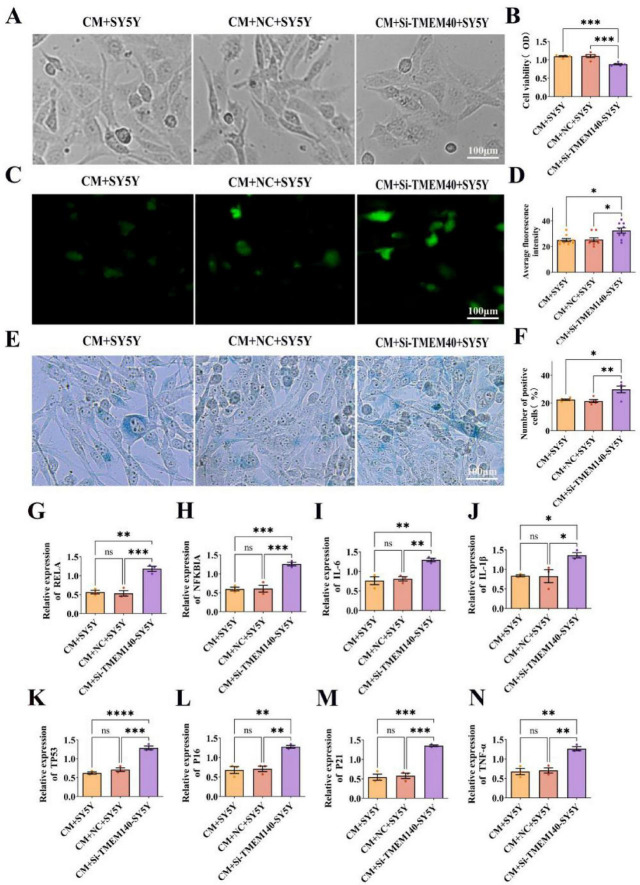
TMEM140 silencing exacerbates PD-conditioned medium-induced injury in SH-SY5Y neuron-like cells. **(A)** Representative morphological images of SH-SY5Y cells under different experimental conditions. **(B)** CCK-8 analysis of cell viability. **(C)** Detection of intracellular ROS levels using the DCFH-DA probe. **(D)** Quantification of ROS fluorescence intensity. **(E)** Representative images of β-galactosidase staining. **(F)** Quantification of the percentage of β-galactosidase-positive cells. **(G–N)** qRT-PCR analysis of expression changes in inflammation-related genes (RELA, NFKBIA, IL-1β, IL-6, and TNF-α) and senescence-related genes (TP53, p21, and p16). Scale bar = 100 μm. *P < 0.05, **P < 0.01, ***P < 0.001, ****P < 0.0001.

To explore the regulatory role of TMEM140 in the transition from peripheral inflammation to central nervous system injury, TMEM140 was silenced in SH-SY5Y neuron-like cells using siRNA, followed by treatment with conditioned medium (CM) generated from HGFs stimulated with the same batch of PG-LPS. The results demonstrated that, under identical inflammatory stimulation conditions, TMEM140 knockdown markedly exacerbated injury in neuron-like cells, characterized by obvious cellular shrinkage and fewer neuritic processes (Figure 12A), and CCK-8 assays further showed a significant reduction in cell viability (*P* < 0.001) (Figure 12B). In parallel, DCFH-DA analysis showed a marked increase in intracellular ROS levels (*P* < 0.05) (Figures 12C,D), while β-galactosidase staining revealed a higher proportion of positive cells (*P* < 0.01) (Figures 12E,F), indicating that reduced TMEM140 expression may intensify oxidative stress and accelerate the cellular senescence process. Further qRT-PCR analysis showed that TMEM140 silencing significantly upregulated RELA, NFKBIA, IL-1β, IL-6, and TNF-α expression (Figures 12G–N), together with increased expression of TP53, p21, and p16, suggesting that loss of TMEM140 may participate in regulating the NF-κB inflammatory pathway and the p53-p21/p16 senescence axis, thereby increasing the susceptibility of neuron-like cells to PD-conditioned medium and exacerbating cellular injury.

## Discussion

### Principal findings and significance

By integrating transcriptomic analysis with *in vitro* validation, this study screened aging-related genes common to PD and AD and indicated that TMEM140 may be a potential molecular bridge connecting the two diseases. The findings demonstrated that TMEM140 tended to be upregulated in PD-related models but was downregulated in Aβ-treated neuron-like cells and cellular senescence models. Additional functional assays indicated that silencing TMEM140 in SH-SY5Y cells exacerbated oxidative stress, inflammatory responses, and senescence-related phenotypes induced by PD-conditioned medium, as reflected by decreased cell viability, increased ROS levels, more SA-β-Gal-positive cells, and enhanced expression of IL-1β, IL-6, TNF-α, p16, p21, RELA, NFKBIA, and TP53. The above findings suggest that decreased TMEM140 expression may be linked to impaired neuronal resistance to inflammatory stress and activation of inflammation-associated senescence pathways. Taken together, from the cross-disciplinary perspective of periodontology and brain aging/AD, this study offers experimental support for a possible link between PD and neurodegenerative changes and highlights TMEM140 as a worthwhile target for further mechanistic studies and potential intervention strategies.

### Peripheral inflammation influences the central nervous system via the immune-neural axis

Chronic peripheral inflammation acting through the immune-neural axis to influence the central nervous system is a major research focus in the fields of aging and neurodegenerative disorders (Nguyen and Cho, 2025; Rim et al., 2024). PD is a chronic infection-related inflammatory disease that may lead to sustained systemic inflammation and immune imbalance (Martínez-García and Hernández-Lemus, 2021). Periodontal pathogens and their virulence products, including *P. gingivalis*, its LPS, and gingipains, may enter the circulation and act on the brain via the blood-brain barrier or neural routes (Gharaibeh et al., 2025; Lundergan et al., 2024). Animal experiments have demonstrated that periodontal pathology can trigger neuroinflammatory responses and cognitive deficits, as mice with tooth loss or PD show hippocampal microglial activation, upregulation of pro-inflammatory cytokine mRNAs including TNF-α, IL-6, and IL-1β, and marked impairment of memory function (Lundergan et al., 2024). Another study reported that blood levels of IFN-β and the interferon-induced protein IFITM3 were increased in PD mice, thereby promoting Aβ deposition and cognitive deficits, which suggests that peripheral inflammation may exacerbate AD-like pathology in the brain through interferon signaling (Kong et al., 2025). This evidence supports the concept of “inflammaging,” whereby aging is accompanied by a chronic, low-grade inflammatory state that renders multiple tissues and organs, including the brain, more vulnerable to inflammatory damage (Kase et al., 2025). For instance, Kase et al. reported that PD mice displayed aging-like systemic inflammatory features (“inflammaging”), together with muscle and skeletal frailty, decreased adult hippocampal neurogenesis, microglial activation, and eventual cognitive deterioration (Kase et al., 2025). Further evidence indicates that PD-induced peripheral inflammation may impair the central nervous system through several mechanisms: first, pro-inflammatory cytokines in blood and tissues, including TNF-α, IL-6, and IL-1β, are markedly elevated, compromising blood-brain barrier (BBB) integrity and enabling inflammatory mediators and immune cells to enter brain tissue; second, these signals can directly activate microglia and astrocytes in the brain, thereby initiating neuroinflammation and cellular senescence responses (Furutama et al., 2020). For example, Furutama et al. observed in a PD mouse model that serum IL-6 was significantly increased after PD, accompanied by upregulation of IL-1β in the hippocampus and reduction of the tight junction protein Claudin-5, indicating disruption of BBB integrity (Furutama et al., 2020). Kase et al. further reported that PD mice exhibited enhanced microglial activation and reduced hippocampal neurogenesis in the brain, resulting in deficits in spatial learning and memory (Kase et al., 2025). Review studies have pointed out that pathogen-derived DNA or inflammatory mediators from PD lesions are frequently detected in the brains of patients with AD, suggesting that oral pathogens and inflammatory signals can access the central nervous system through hematogenous or neural routes (Wang et al., 2019). This process is tightly linked to “inflammaging,” as immunosenescence leads to sustained release of peripheral inflammatory mediators and senescence-associated secretory phenotype (SASP) factors such as IL-6 and MMPs, thereby driving functional deterioration across multiple organs (Nguyen and Cho, 2025; Rim et al., 2024). At the same time, senescent microglia and astrocytes within the brain also release SASP factors, which maintain chronic neuroinflammation and functional dysfunction (Nguyen and Cho, 2025; Rim et al., 2024). Taken together, chronic peripheral inflammation caused by PD interacts with immune senescence in the brain and induces neuroinflammatory responses through the immune-neural axis, ultimately accelerating brain aging and the progression of neurodegenerative pathology.

### The role of TMEM140 in immune regulation and cellular senescence

TMEM140 is a transmembrane protein whose involvement in immune-inflammatory processes is a relatively recent discovery. Earlier studies have primarily investigated TMEM140 in oncology and autoimmune diseases, showing that it is highly expressed in glioma and promotes tumor cell proliferation and invasion (Schmit and Michiels, 2018). In gene network analyses of comorbidity between systemic lupus erythematosus and PD, TMEM140 was identified as a key hub gene (Cheng et al., 2025). These results indicate that TMEM140 may participate in modulating immune responses and cellular proliferation. In the present study, TMEM140 appeared to have a bidirectional regulatory role in peripheral inflammation and neuroaging. Integrated transcriptomic data analysis revealed that TMEM140 was strongly associated with immune cell infiltration. In AD tissue, TMEM140 showed a significant negative correlation with plasma cell infiltration and a positive correlation with γδ T-cell infiltration. In PD, increased TMEM140 expression was associated with alterations in various immune cell types, including neutrophils. These findings suggest that TMEM140 may shape the course of peripheral immune responses. For instance, TMEM140 expression was elevated in gingival fibroblasts following PG-LPS treatment, indicating that TMEM140 may participate in regulating local periodontal inflammation. In the *in vitro* model, TMEM140 was silenced in SH-SY5Y cells to mimic its reduced regulation under PD-related conditions, and the results showed that TMEM140 knockdown exacerbated the injury induced by PD-conditioned medium in neuron-like cells. This was manifested by a further reduction in cell viability and increases in oxidative stress levels and the proportion of SA-β-Gal-positive cells. Consistent with this, neuronal cells in the TMEM140-silenced group showed significant upregulation of the pro-inflammatory cytokines IL-1β, IL-6, and TNF-α, along with marked increases in the cell cycle inhibitors p16 and p21, suggesting that reduced TMEM140 expression enhances activation of inflammatory signaling and senescence pathways. These findings imply that TMEM140 plays a critical role in immune regulation and inflammatory negative feedback, and that its normal expression may help restrain the propagation of inflammatory responses and prevent excessive neuronal senescence. In integrated transcriptomic analyses, TMEM140 was identified as a shared core gene in PD and AD along with other aging-related genes such as TIMP1 and ALDH2, but its opposite expression patterns in the two pathological contexts underscore its unique functional role (Yang et al., 2024). Taken together, TMEM140 may play a pivotal role in PD-driven brain aging by modulating peripheral inflammatory intensity and immune cell activity, while also affecting neuronal ROS levels and senescence-related signaling.

### Comparison with previously reported bridging factors

A number of bridging factors have previously been reported to participate in the link between PD and brain aging. The classical pro-inflammatory mediators IL-6 and TNF-α are significantly elevated in patients with both PD and AD and are capable of inducing neuroinflammation (Furutama et al., 2020). For instance, increased IL-6 induced by PD can reach brain tissue through the bloodstream, resulting in elevated IL-1β expression in the hippocampus and neuronal damage (Furutama et al., 2020). IL-1β and TNF-α within the brain are likewise thought to be involved in cognitive dysfunction. Moreover, TREM2, a receptor specific to microglia, is a key regulator of innate immune function, and mutations in TREM2 markedly increase AD risk, highlighting the importance of microglial phagocytic and inflammatory regulatory pathways in AD pathogenesis (Liccardo et al., 2020). Senescence-associated SASP molecules also serve as bridging factors; for example, TIMP1 and other SASP factors secreted by astrocytes may influence neuronal survival and extracellular matrix remodeling. ALDH2, a cellular metabolic enzyme involved in oxidative stress regulation, may exacerbate neuroinflammation and accelerate cognitive decline when deficient (Joshi et al., 2019). By contrast, the present study suggests that TMEM140, as a novel transmembrane protein factor, may operate through a mechanism distinct from those of the aforementioned factors. Rather than acting as a single secreted molecule or receptor, it may mediate the transmission of PD-derived inflammatory signals to the brain through novel signaling pathways, such as those involving cell-cell adhesion and inflammatory cascades. This finding offers a new perspective on the mechanisms underlying PD-associated brain aging, suggesting that in addition to established cytokine and metabolic pathways, membrane protein-mediated regulatory networks may also be involved.

### Comparison with prior studies

Earlier studies have shown that several bridging factors are involved in the association between oral inflammation and brain aging/AD. For instance, PD-related systemic inflammation is accompanied by marked increases in circulating pro-inflammatory cytokines, including IL-1β, IL-6, and TNF-α (Lundergan et al., 2024). These cytokines may traverse the blood-brain barrier, activate microglia and astrocytes in the brain, and thereby facilitate Aβ production and neuronal damage. Moreover, TREM2, a broadly recognized inflammation-related factor, is regarded as an important regulator of immune responses in AD. TIMP1, a matrix metalloproteinase inhibitor, has been implicated in both periodontal tissue destruction and central inflammation, and is involved in regulating tissue repair during systemic inflammatory responses (Yang et al., 2024). With respect to ALDH2, clinical and model-based studies have demonstrated that ALDH2 deficiency exacerbates Aβ pathology (Wang et al., 2024), and our study likewise identified ALDH2 as a shared gene in PD and AD. In comparison with these established mechanisms, the TMEM140-related mechanism proposed in this study appears to be novel. TMEM140 is not a classical inflammatory mediator or enzyme, but instead is involved in membrane-associated signaling transduction. Instead of acting through direct cytokine release, TMEM140 may indirectly modulate the intensity of inflammation by regulating immune cell infiltration and the activity profile of glial cells. This is somewhat distinct from the conventional view and offers a new perspective, namely that transmembrane proteins in peripheral tissues may function as negative feedback regulators that mitigate chronic inflammation-induced brain injury. Overall, this study not only confirms earlier findings that common PD-associated pro-inflammatory factors (such as IL-6 and TNF-α) are linked to brain aging, but also identifies TMEM140 as a novel regulatory node, suggesting that the pathogenesis of AD may involve additional complex and previously underappreciated transmembrane signaling pathways.

### Limitations of the study

Although the present study indicates that PD-related inflammation may influence brain aging through the peripheral-central immune network and suggests a potential bridging role for TMEM140, several limitations should be acknowledged. First, the current findings are primarily derived from bioinformatics analyses and *in vitro* experiments, and direct evidence from *in vivo* TMEM140 knockout, overexpression, or pharmacological intervention is still lacking, so its causal role cannot yet be confirmed. Second, integrated transcriptomic analysis can identify candidate pathways and potential mechanisms, but it cannot directly establish causal relationships among molecules; therefore, further verification using animal models, time-course analyses, and functional rescue experiments is still needed. Moreover, single-cell analysis indicated that TMEM140 is predominantly enriched in oligodendrocytes and microglia, whereas the functional experiments in this study were mainly conducted in SH-SY5Y neuron-like cells, and therefore may not adequately represent its specific functions across different brain cell types. Finally, the present study primarily focused on inflammatory and aging-related phenotypes at fixed time points and did not systematically assess the dynamic relationship between PD progression and brain pathological alterations; these issues warrant further investigation in future studies.

### Future directions and clinical translation

This study points to new directions for the future prevention and intervention of PD-associated brain aging. On the one hand, therapeutic strategies targeting TMEM140 itself or its downstream pathways may be explored, such as screening for TMEM140 inhibitors or modulators. On the other hand, currently available anti-inflammatory strategies could also be applied to mitigate the subsequent effects of PD. For instance, IL-6 or TNF-α inhibitors are already used in several inflammatory disorders, and their potential to prevent the propagation of peripheral inflammatory signals to the brain warrants investigation. In addition, interventions aimed at aging-related mechanisms also appear promising; ALDH2 activators such as Alda-1 have been shown in mouse models to reduce oxidative brain damage and cognitive decline (Joshi et al., 2019). Early clinical trials of senolytic therapies used in combination in patients with Alzheimer’s disease have shown reductions in inflammatory biomarkers in the cerebrospinal fluid (Shafqat et al., 2023). These strategies, when integrated with PD treatment, may synergistically slow brain aging. TMEM140 and related inflammatory markers may also serve as early warning biomarkers for monitoring inflammation-associated aging through blood or cerebrospinal fluid testing. Epidemiological studies have reported that patients with severe PD are at a significantly higher risk of cognitive decline (Igase et al., 2024). Future prospective cohort studies may validate the relationship between TMEM140 and other markers and cognitive deterioration, and integrate these indicators into risk prediction models. In conclusion, this study opens a new avenue for research into PD and brain aging, provides scientific support for periodontal health management and early intervention in AD, and further suggests the development of dual-target interventions against inflammation and aging, such as combining oral or local anti-inflammatory treatment with AD therapeutics or investigating whether improved periodontal health protects cognitive function, thereby offering novel diagnostic and therapeutic strategies for the intersection of PD and neuroaging (Bowirrat, 2022; Lundergan et al., 2024).

## Data Availability

Publicly available datasets were analyzed in this study. This data can be found here: GSE10334; GSE223924; GSE16134; GSE122063; GSE132903; GSE110226.
